# Potential of Hedgerows with Aromatic Plants as Reservoirs of Natural Enemies of Pests in Orange Orchards

**DOI:** 10.3390/insects14040391

**Published:** 2023-04-17

**Authors:** Theodoros Stathakis, Leonidas Economou, Myrto Barda, Theodoros Angelioudakis, Vaya Kati, Filitsa Karamaouna

**Affiliations:** Scientific Directorate of Pesticides Control and Phytopharmacy, Benaki Phytopathological Institute, 8 Stefanou Delta Str., 14561 Kifissia, Greece

**Keywords:** aromatic plants, conservation, natural enemies, oregano, parasitoid, predator, rosemary, sage, savory, weed flora

## Abstract

**Simple Summary:**

The present study examined the ability of four Mediterranean aromatic plant species, i.e., oregano, rosemary, sage, and savory, established in hedgerows in orange orchards to support natural enemies of citrus pests in comparison to margins with bare soil or weed vegetation, which is the commonly used management practice. The impact of the aromatic plant hedgerows on conservation of parasitoid wasps, spiders, and predators of insect pests varied with the aromatic plant species and natural enemy group, i.e., savory plants had a positive effect on the abundance of parasitoids and arachnid predators; sage and oregano flowers favored predatory insects, but sage harbored low numbers of parasitoids compared to the main flowering weed species; rosemary hedgerows served as a most suitable habitat for spiders in comparison to the pre-existing weed cover. The results support the use of the tested aromatic plant species as customized reservoirs for certain groups of beneficial arthropods in orange orchards, considering also the possible incorporation of suitable flowering wild plant species in the weed flora.

**Abstract:**

In the present study, the potential of hedgerows from Mediterranean aromatic plant species, i.e., oregano, rosemary, sage, and savory, in orange field margins to function as reservoirs of natural enemies of citrus pests was tested in comparison to the common management practice of bare soil or weed vegetation. Assessments were based on the abundance and diversity of parasitoid wasps, spiders, and insect predators in the field margins and on the orange trees for two growing seasons. Savory plants harbored more parasitoids compared to weed vegetation and the other aromatic plants (savory > organic rosemary > sage > oregano). Weed vegetation hosted more arachnid predators than the aromatic plants in their first year in the orchard, but this was reversed with their full growth in the following year (most abundant on rosemary). Oregano and sage favor insect predators. The similarity of the natural enemy communities on the field margins and on the orange trees increased with time, indicating the insects’ movement from the field margins to the trees. The results support the use of the tested aromatic plant species in conservation practices for targeted groups of beneficial arthropods in orange orchards, also considering the exploitation of suitable wild flowering plants of the weed flora.

## 1. Introduction

In recent decades, rising demands for agricultural products led to the displacement of traditional cultivation systems by intensive and specialized farming systems, which rely on external inputs of agrochemicals and energy [1]. Moreover, intensive monocrop systems are responsible for biodiversity loss, water pollution, high greenhouse gas emissions, soil degradation, and reductions in ecosystem services [2].

Landscape elements such as hedges, grassland, wildflower strips, field margins, and headlands are ecological infrastructures, whose judicious use increases the functional biodiversity of the farm by providing habitats for aestivation or overwintering [3], breeding sites [4], as well as food resources such as nectar and pollen [5,6], and alternative prey/host for parasitoids or pest predators [7,8]. Thus, they reduce the need for pesticide applications and support an appropriate environment protected from agricultural operations [9,10,11,12,13,14,15].

Conservation biological control (CBC) is a sustainable approach in pest management that can contribute to a reduction in pesticide use as part of an Integrated Pest Management (IPM) strategy by supporting populations of natural enemies present in the agroecosystem and by promoting their effectiveness as predators, parasitoids, or pathogens [16,17,18,19]. This strategy is especially useful in permanent evergreen crops [9,20], such as citrus, where both pests and their natural enemies are active and abundant throughout the year [21].

Citriculture is one of the pillars of Mediterranean agriculture, being the dominant citrus fruit producing area worldwide [22]. In Greece, citrus orchards cover an area of about 40,000 ha, representing 43% of total fruit crops [23]. Major arthropod pests in terms of importance in the citrus-growing regions of the country comprise the Mediterranean fruit fly *Ceratitis capitata* (Wiedemann) (Diptera: Tephrididae), the California red scale *Aonidiella aurantii* (Maskell) (Hemiptera: Diaspididae), and the mealybug *Planococcus citri* (Risso) (Hemiptera: Pseudococcidae). However, outbreaks of the woolly whitefly *Aleurothrixus floccosus* Maskell (Hemiptera: Aleyrodidae), the citrus whitefly *Dialeurodes citri* (Ashmead) (Hemiptera: Aleyrodidae), *Ceroplastes rusci* L (Hemiptera: Coccidae), the Mediterranean black scale *Saissetia oleae* (Bernard) (Hemiptera: Coccidae), and Tetranychidae and Eriophyiidae mites are recorded at small (local) scale. The citrus leafminer *Phyllocnistis citrella* (Stainton) (Lepidoptera: Gracillariidae) and the citrus infesting aphids and thrips are of minor importance [24]. Recently, the citrus spiny whitefly, *Aleurocanthus spiniferus* (Hemiptera: Aleyrodidae), an economically important species, was added to the citrus pest list [25].

Conservation biological control has been proven very effective in the management of insect pests in citrus orchards, including ground cover management practices and the use of banker plants [26]. Wind breaks or hedgerows of *Nerium oleander* L. (Apocynaceae) serve as a reservoir for aphid natural enemies [26]; conservation of weeds such as *Oxalis pes-caprae* L. (Oxalidaceae) provides non-pest spider mites as alternative prey for phytoseiid mites [27]; sown cover of *Festuca arundinacea* Schreb. (Poaceae) regulates populations of *Tetranychus urticae* Koch (Tetranychidae) [28,29,30,31]; and enhances ground-dwelling predators of *C*. *capitata* [32].

In this context, a diversified farming system combining citrus culture, aromatic medicinal plants, and apiculture is tested within the frame of the PRIMA project, with the acronym PLANT-B, which aims to make the best use of resources for the benefit of citrus fruit and honey production in the Mediterranean region. In particular, this farming system exploits field margins as semi-natural habitats for pollinators and natural enemies to enhance pollination and biological control services and subsequently improve the quality, safety, and security of citrus and honey produce.

Insectary plants, by definition, include flowering plants that attract and possibly maintain, with their nectar and pollen resources, a population of natural enemies that contribute to biological pest management on crops [33]. The establishment of selected flowering plant species in field margins to support beneficial arthropods for pest control and crop pollination is a widely adopted practice [6,34,35,36,37,38,39,40]. Most of these attempts concern mixes of annual flower species in various crops, e.g., tomato, watermelon, apple, and olive [41,42,43,44,45]. Aromatic plants do not represent a homogeneous taxonomic group but are defined by their chemical properties, in particular volatile compounds (terpenoids, steroids, alkaloids, and organic cyanides) that correspond to olfactory attributes that may be used in medicine, food, or plant protection [46,47,48,49]. They also have the agronomic advantage of being potentially marketable compared to other wildflower species [50]. However, their suitability to serve as insectary plants in citrus production is undetermined.

The aim of the present study was to assess the ability of four Mediterranean perennial aromatic plant species, namely oregano, rosemary, sage, and savory, established in field margins to attract and support natural enemies of citrus pests in comparison with the commonly used management practice of bare soil or weed vegetation. The study also investigates arthropod diversity in such environments (aromatic plants vs. weed vegetation) as well as on trees in orange orchards with the respective field margin management.

## 2. Materials and Methods

### 2.1. Experimental Fields and Establishment of Flowering Hedgerows

**Figure 1:**
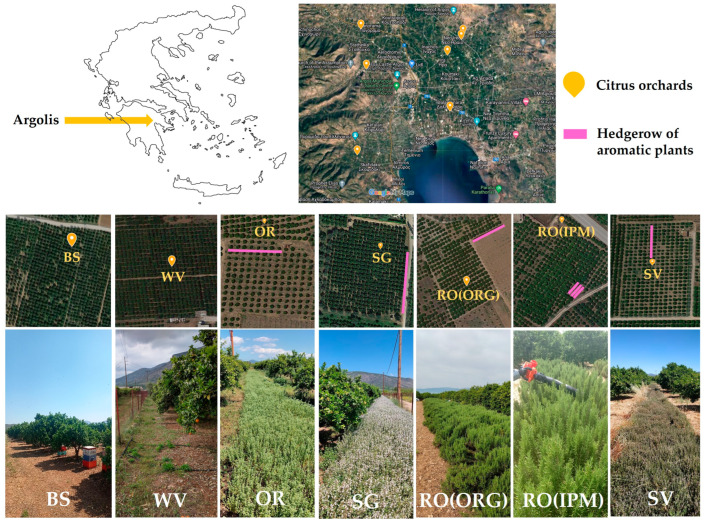
Experimentation sites of seven orange orchards in Argolis, Peloponnese, Greece, and layout of the aromatic plant hedgerows. [pink lines and photographs OR, SG, RO(ORG), RO(IPM), SV] and the control fields [weed vegetation (WV) and bare soil (BS)]. RO(ORG): rosemary hedgerow (organic orchard); RO(IPM): rosemary hedgerow (IPM orchard); OR: oregano hedgerow; SG: sage hedgerow; SV: savory hedgerow.

The study was carried out in seven orange orchards (five with cv. Navelina and two with cv. Washington Navel) in Argolis, Peloponnese, Greece, during three consecutive growing seasons (2020, 2021, and 2022) (Figure 1). A 0.5 ha study area was delineated in each orchard, with 30–45-year-old trees of the goblet cultivation system. Pest management in all orchards used IPM principles except for one orchard which was organic. The plant protection interventions for each orchard are shown in Supplementary Table S1.

The first year (2020) was a baseline year, i.e., no intervention was made in the field margins. In the second year (2021), one aromatic species was established as a hedgerow in one field margin in five orchards, whereas one field margin in either of the two other orchards was used as a control, i.e., bare soil with no vegetation (BS) and weed vegetation (WV). Four aromatic plant species of the family Lamiaceae were tested: rosemary (*Rosmarinus officinalis* L.) in one IPM field [RO(IPM)] and the organic field [RO(ORG)], sage (*Salvia officinalis* L.) (SG), savory (*Satureja thymbra* L.) (SV), and oregano (*Oreganum vulgare* L.) (OR). The aromatic plant hedgerows covered an area of 80–120 m^2^, depending on the available field margin, with an average width of 2 m. The height of each hedgerow ranged as follows: RO(IPM) 80–100 cm, RO(ORG) 100–120 cm, SG 30–90 cm, SV 30–40 cm, and OR 30–60 cm on their first and second years in the field margins, respectively. In all orchards during the baseline year and in the control fields during the next two years, the growers followed the usual agricultural practices regarding weed management in the field margins. Regarding weed management in the orange orchards, 2–4 weed mowing treatments were performed per year between the tree rows. In addition, in all orchards except for the RO(ORG) and SG, two herbicide applications (most commonly glyphosate) were performed along the tree rows or on a larger groundcover area (BS orchard) using a small boom sprayer (Supplementary Table S1). Further targeted herbicide applications were carried out by the farmers with a backpack sprayer when needed.

The experimental design included seven field margin treatments: hedgerows of five different aromatic plant species, weed vegetation, and bare soil. In each orchard, the effect of the field margin management on the presence of natural enemies was measured in the field margins (3 randomly selected plots of 10 m^2^ in the hedgerows or in the field margins with weed vegetation) and on the canopy of three orange trees next to the tested field margin and in the fifth row from the field margin (Figure 1). Thus, in each orchard, three replications were made in the field margins and three replications on the tree canopy in each of the two row (next to the field margin, five rows from the field margin).

### 2.2. Flower and Plant Cover Measurements in Field Margins

Visual estimation of plant and flower cover percentages (%) were performed on plots with aromatic plants (orchards: RO(IPM), RO(ORG), (SG), (SV), and (OR)) or weeds in the field margins (all orchards in the baseline year, WV orchard the following two years). These measurements were performed on the insect sampling dates. The methodology followed approaches described in [42,43,44] and was adapted for the experimental design of the current study. More specifically, the plant and flower covers were visually estimated and expressed as percentages of the plot area (10 m^2^) in all examined plots. In the case of weed flora, total and per-species flower cover were estimated to determine the main flowering weed species present in the respective plots. Flowering plant species were identified in situ or, when necessary, collected and identified in the laboratory using the botanical identification keys Flora Europaea [51,52,53,54,55].

### 2.3. Arthropod Presence Measurements

Measurements of the arthropod presence (abundance, diversity) in the field margins and on the orange trees were performed in the spring of each experimental year (2020–2022), during and after the orange tree blossom (BBCH 60-70) and aromatic plants’ flowering. Measurements were not taken in orchards that had null vegetation in their margins (BS throughout the experimental period; RO(ORG) and OR in baseline year 2020). To collect data on beneficial arthropods, suction sampling using a modified leaf blower was conducted as described in [42,43,44]. In the field margins, eight random suctions of two seconds per suction were performed in each plot (16 s/10 m^2^). On the tree canopy, eight random suctions of two seconds per suction (16 s/tree) were made, 2 in each cardinal point, at 2 m above the soil surface, from 3 trees next to the filed margins and from 3 trees in the fifth row.

The arthropod samples were kept in the freezer (−18 °C) and sorted by family, genus, and species (where possible) under a stereomicroscope using taxonomic keys [56,57,58]. The identified arthropods were classified into three major functional groups: Hymenoptera parasitoids (25 families), Arachnid predators (16 spider families and harvestmen), and insect predators (10 families: Dermaptera and Mantodea) (Supplementary Table S2).

### 2.4. Statistical Analysis

The effect of field margin management (aromatic plants, hedgerows, weed vegetation, and bare soil) on the attraction of natural enemies of pests was determined using one-way ANOVA (α = 0.05) on the data from the treated orchards (BS, WV, RO(ORG), RO(IPM), OR, SG, SV) for each experimental year separately. When the effect was significant, the means were separated using Tukey’s HSD test (α = 0.05). Data on the number of beneficial arthropods (parasitoids, arachnids, and insect predators) were transformed (log(x + 1)) to achieve a better fit to the assumptions of the analyses (homoscedacidity). The statistical analyses were performed using the statistical package JMP [59]. The similarity between communities of natural enemies on field margins and orange trees was assessed by Principal Coordinate Analysis (PCoA) and one-way ANOSIM (α = 0.05), with the Bray–Curtis similarity index, using PAST [60].

## 3. Results

### 3.1. Baseline Year

Summarizing the weed vegetation profile in the field margins of the orchards during the baseline year in terms of plant cover, flower cover, and species, this is as follows:

WV field: the plant cover was 45–55% in late April and the respective flower cover was 10–25%; the main weed species recorded were *Crepis* sp. (Asteraceae) and fewer plants of *Anagallis arvensis* L. (Primulaceae).

SG field: in late April, the plant cover was 75–95% with flower cover 60–80%, and the main weed species were *Matricaria chamomilla* L. (Asteraceae) and *Raphanus raphanistrum* L. (Brassicaceae); in early May, plant cover and flower cover were 70–95% and 40–65%, respectively, with the same main weed species as in late April with the addition of *Malva* sp. (Malvaceae).

SV field: the plant cover was 80–100% with the respective flower cover of 20–60% in late April, mainly consisting of *Capsella bursa-pastoris* L. (Brassicaceae), *Veronica persica* Poir. (Plantaginaceae), *Stellaria apetala* (Caryophyllaceae), and *Rapistrum rugosum* L. (Brassicaceae); in early May, the flower cover was 1–10%, mainly consisting of *R*. *rugosum*.

RO (IPM): in late April, the plant cover was 95–100%, and the flower cover was 70–75%, while in early May, the plant and flower covers were 55–100% and 10–45%, respectively. In both periods, the main weed species were *Medicago polymorpha* L. (Fabaceae), *Crepis* ap., *Campanula patula* L. (Campanulaceae), and *S. apetala*.

**Figure 2:**
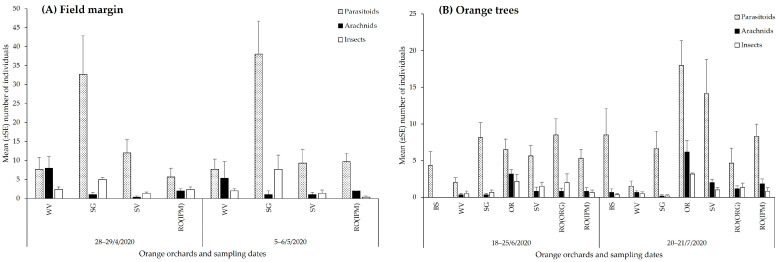
Temporal distribution of natural enemies (parasitoids, arachnid predators, and insect predators) (**A**) in field margins and (**B**) on orange trees at the baseline year (field margin management using the usual farmers’ practice).

Among the four orchards examined, the highest number of parasitoid wasps (Hymenoptera) was observed in the field margin of the SG orchard and the lowest in those of the RO(IPM) and WV (Figure 2A, Supplementary Table S3). On orange trees, the parasitoid abundance in all seven orchards did not differ significantly in the first sampling date (June), while in the second (July), a higher number of parasitoids was collected from the OR orchard, followed by the SV orchard (Figure 2B, Supplementary Table S4).

Arachnid predators were more abundant in the margins of the WV orchard and on orange trees in the OR orchard on all sampling dates. A higher number of predatory insects was collected from the margins of the SG orchard and from orange trees in the OR orchard (Figure 2, Supplementary Tables S3 and S4).

**Figure 3:**
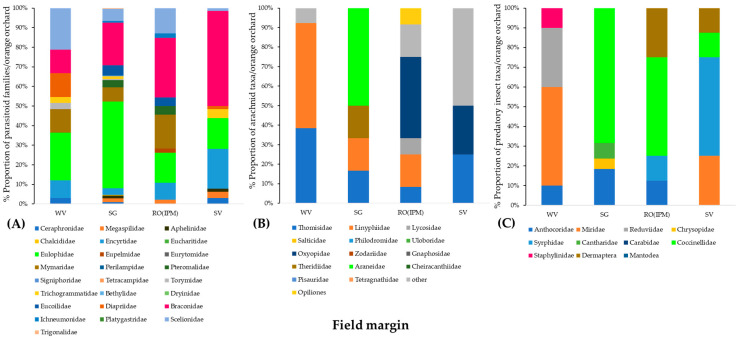
Community composition of (**A**) Hymenoptera parasitoids, (**B**) arachnid predators, and (**C**) insect predators in four orange orchards (WV, SG, SV, and RO(IPM)) at the baseline year (field margin management using the usual farmers’ practice).

Regarding the diversity of natural enemy communities in the field margins of the orchards, the SG orchard had the most diverse community of parasitoid wasps, including 16 different families. Eulophidae and Braconidae were the dominant families in the field margins of the four orchards. Scelionids were more abundant in the WV field margin than Encyrtidae in the SV margin. Mymaridae were found in all field margins except for the SV orchard (Figure 3A).

The spider family Thomisidae was present in all field margins. Linyphiidae had the highest proportion in the WV orchard and was not observed in the SV orchard. Oxyopids were collected only from the RO(IPM) and SV orchards. Web-builders Araneidae and Theridiidae were observed only in the margins of the SG orchard (Figure 3B).

Coccinellidae were present in all field margins except for that of the WV orchard. Predatory mirids were dominant in the WV and SV orchards. Anthocoridae were absent from samples of the SV orchard. Dermaptera and Syrphidae were present only in the field margins of the RO(IPM) and SV orchards (Figure 3C).

**Figure 4:**
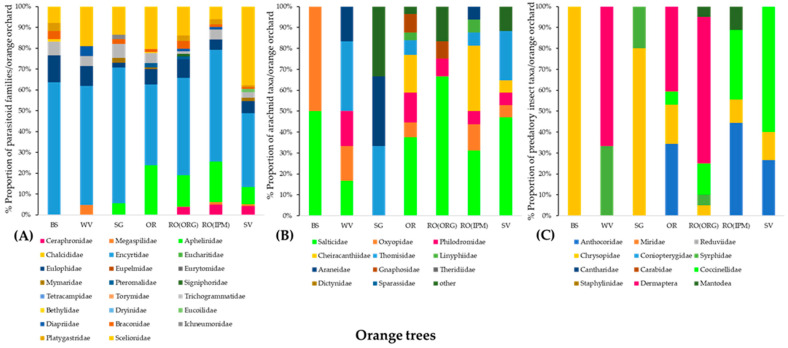
Community composition of (**A**) Hymenoptera parasitoids, (**B**) arachnid predators, and (**C**) insect predators on orange trees in seven orchards (BS, WV, SG, OR, SV, RO(ORG), and RO(IPM)) at the baseline year (field margin management using the usual farmers’ practice).

On orange trees, the dominant parasitoid family was Encyrtidae in all orchards. Scelionids hold a large proportion of all orchards. Eulophidae and Trichogrammatidae were present in all orchards but in low proportions. Aphelinids were collected from orange trees in all fields except the BS and WV. Braconidae individuals were not observed in samples from the tree canopies of the WV orchard (Figure 4A).

From the arachnid predators, Salticidae was the dominant spider family in all orchards except for the SG. Thomisids were absent only from the BS and RO(ORG) orchards, while Oxyopidae were from SG and RO(ORG). Araneidae were collected from the WV, SG, and RO(IPM) fields, and Cheiracanthiidae only from orange trees in the OR, RO(IPM), and SV orchards (Figure 4B).

Chrysopidae were present in all orange orchards except for WV, while this is the only predatory insect taxon collected from the BS orchard. Coccinellids were collected from the OR, RO(ORG), RO(IPM), and SV orchards. The same applies to Anthocoridae bugs except for the RO(ORG) orchard. Predatory syrphids were present on orange trees in WV, SC, and RO(ORG) orchards, while omnivore Dermaptera were present on those in WV, OR, and RO(ORG) orchards (Figure 4C).

### 3.2. After the Establishment of Hedgerows

#### 3.2.1. Spatial and Temporal Patterns of Natural Enemies

**Figure 5:**
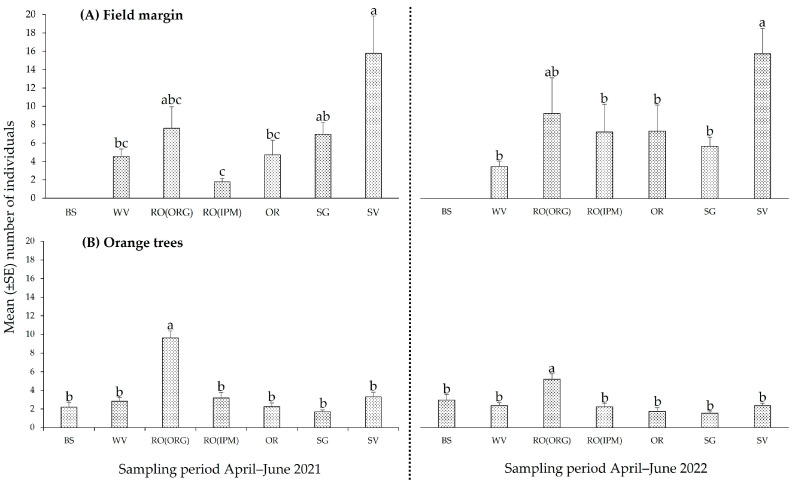
Total abundance (mean ± SE) of Hymenoptera parasitoids (**A**) in field margins and (**B**) on orange trees in orchards with different field margin management: BS—bare soil; WV—weed vegetation; RO(ORG)—rosemary hedgerow (organic orchard); RO(IPM)—rosemary hedgerow (IPM orchard); OR—oregano hedgerow; SG—sage hedgerow; SV—savory hedgerow. Identical letters above the error bar indicate no statistically significant differences among orchards.

Over time, the total abundance of natural enemies varied to a considerable extent, both on the aromatic plants and on the orange trees. In the field margins, the highest number of parasitoid wasps (Hymenoptera) was harbored by the SV orchard in both sampling periods (2021: *F*_5,102_ = 6.3278, *p* ˂ 0.001, 2022: *F*_5,120_ = 6.3895, *p* ˂ 0.001) (Figure 5A), while on orange trees, the highest number of Hymenoptera parasitoids was collected from the RO(ORG) orchard in both sampling periods (2021: *F*_6,245_ = 19.7607, *p* ˂ 0.001, 2022: *F*_6,287_ = 7.5434, *p* ˂ 0.001) (Figure 5B).

**Figure 6:**
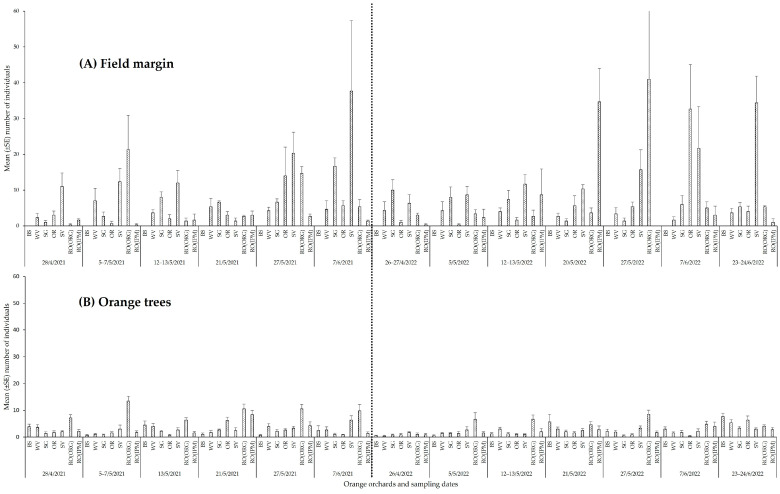
Temporal distribution of Hymenoptera parasitoids (**A**) in field margins and (**B**) on orange trees in orchards with different field margin management: BS—bare soil; WV—weed vegetation; RO(ORG)—rosemary hedgerow (organic orchard); RO(IPM)—rosemary hedgerow (IPM orchard); OR—oregano hedgerow; SG—sage hedgerow; SV—savory hedgerow.

Looking at their temporal abundance fluctuation, parasitoid wasps on SV reached peak levels from late May to early June 2021 and similarly attained their highest number in June 2022. On RO(ORG), a population peak was observed in early May 2021, while population peaks were recorded in both RO(ORG) and (IPM) orchards from late May to June 2022. The population pattern of parasitoid wasps on OR was similar to that of RO hedgerows in 2022 (Figure 6A, Supplementary Tables S5 and S7). Parasitic wasps on orange trees in the rosemary RO(ORG) orchard had significantly higher abundance levels compared to the other orchards on all sampling dates in both years. In the other orchards, population peaks were observed in OR, RO(IPM), and SV fields from late May to June in 2021, while peaks were recorded in BS and OR fields in late June in 2022 (Figure 6B, Supplementary Tables S6 and S8).

**Figure 7:**
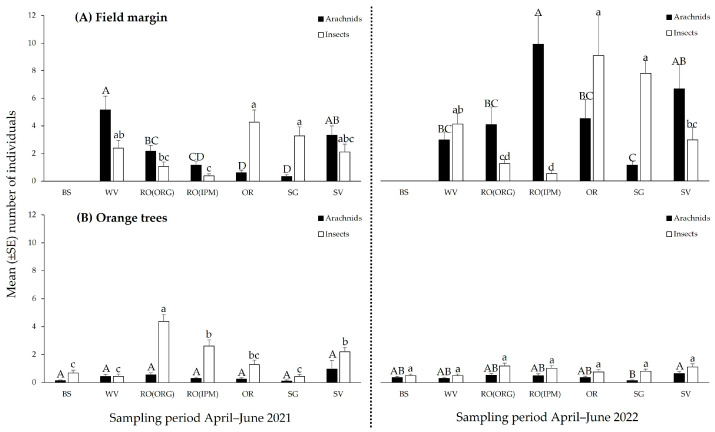
Total abundance (mean ± SE) of arachnid and insect predators (**A**) in field margins, (**B**) on orange trees in orchards with different field margin management: BS—bare soil; WV—weed vegetation; RO(ORG)—rosemary hedgerow (organic orchard); RO(IPM)—rosemary hedgerow (IPM orchard); OR—oregano hedgerow; SG—sage hedgerow; SV—savory hedgerow. Identical letters above the error bar indicate no statistically significant differences among orchards.

**Figure 8:**
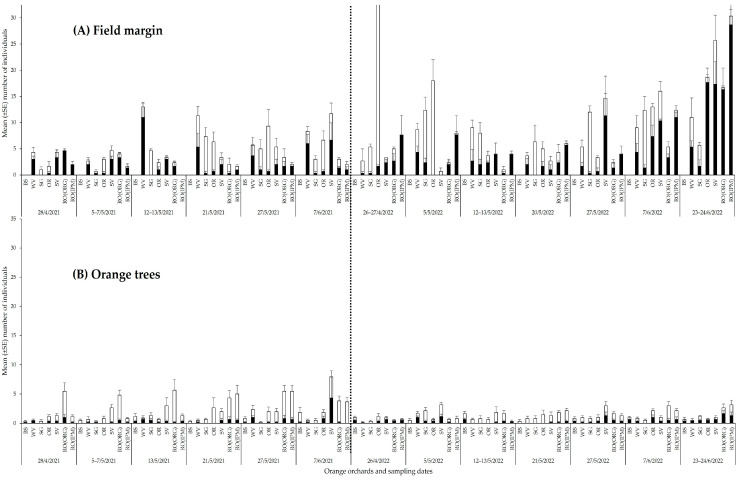
Temporal distribution of arachnid and insect predators (**A**) in field margins, (**B**) on orange trees in orchards with different field margin management: BS—bare soil; WV—weed vegetation; RO(ORG)—rosemary hedgerow (organic orchard); RO(IPM)—rosemary hedgerow (IPM orchard); OR—oregano hedgerow; SG—sage hedgerow; SV—savory hedgerow.

In 2021, the highest number of arachnid predators was observed in the field margin of the WV orchard (*F*_5,102_ = 16.2965, *p* ˂ 0.001) with a peak in mid-May, while in 2022 arachnids were most abundant on the RO(IPM) hedgerow (*F*_5,120_ = 7.0375, *p* ˂ 0.001) with a peak in June (Figure 7A and Figure 8A, Supplementary Tables S5 and S7). Orange trees in the SV orchard harbored the highest number of arachnids in both sampling periods (2021: *F*_6,245_ = 2.2569, *p* = 0.0387; 2022: *F*_6,287_ = 2.2058, *p* = 0.0426) (Figure 7B). In 2021, the population did not differ between sampling dates except for a peak in the SV orchard in June. In 2022, arachnids on the orange trees reached their highest abundance in the SV orchard during May but in the RO(ORG) and RO(IPM) orchards in late June (Figure 8B, Supplementary Tables S6 and S8).

The highest numbers of predatory insects were observed in the field margins with OR and SG in both sampling periods (2021: *F*_5,102_ = 7.5062, *p* ˂ 0.001, 2022: *F*_5,120_ = 16.6819, *p* ˂ 0.001) (Figure 7A). The orchard that harbored the highest number of predatory insects on the orange trees in both sampling periods was the RO(ORG), followed by the RO(IPM) and SV orchards (2021: *F*_6,245_ = 20.2596, *p* ˂ 0.001, 2022: *F*_6,287_ = 2.5846, *p* = 0.0187) (Figure 7B). In 2021, the period of the highest abundance of predatory insects on OR and SG hedgerows was late May, while in 2022 the highest abundance on OR was late April to early May but on SG it was late May to June (Figure 8A, Supplementary Tables S5 and S7). On orange trees, the significantly higher abundance of the insect predators in the RO(ORG) orchard lasted almost the whole sampling period in 2021, while population peaks were observed in the RO(IPM) and SV orchards from late May to June. In 2022, predaceous insects on orange trees showed significantly higher abundance in the SV orchard early in May than in the RO(ORG) orchard early in June (Figure 8B, Supplementary Tables S6 and S8).

#### 3.2.2. Community Composition of Natural Enemies

**Figure 9:**
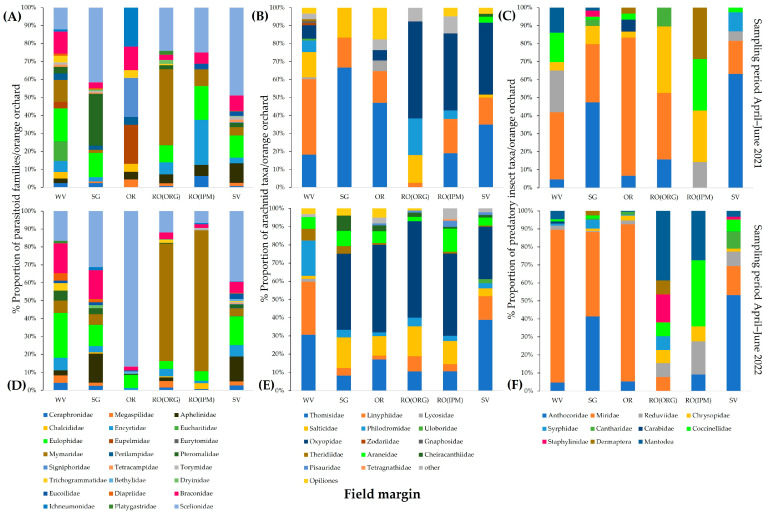
Community composition of natural enemies in field margins of orange orchards with different field margin management. Sampling period April–June 2021: (**A**) Hymenoptera parasitoids; (**B**) arachnid predators; (**C**) insect predators. Sampling period April–June 2022: (**D**) Hymenoptera parasitoids; (**E**) arachnid predators; (**F**) insect predators. WV—weed vegetation; RO(ORG)—rosemary hedgerow (organic orchard); RO(IPM)—rosemary hedgerow (IPM orchard); OR—oregano hedgerow; SG—sage hedgerow; SV—savory hedgerow.

The community structure of beneficial arthropods varied among the different field margins of aromatic plants and weed vegetation. In 2021, the most diverse community of parasitic Hymenoptera was observed on WV, represented by 17 different families, followed by SV (15 families), while the least diverse was on RO(IPM) (8 families). Scelionidae was the dominant family in all field margins, except for OR, especially on SV and SG (41.60 and 48.94%, respectively). Mymaridae had a high proportion (42.34%) on RO(ORG). In 2022, greater diversity of parasitic wasps was observed on SV (19 families), followed by SG (14 families). Scelionids were abundant in almost all field margins, but, unlike in 2021, they were super dominant in OR (86.84%). Mymarids had the highest proportions on RO(ORG) and RO(IPM) (65.63% and 78.67%, respectively) (Figure 9A,D).

The most diverse composition of arachnid predator taxa during 2021 was found in WV (11) and the least in SG (3). Thomisidae and Linyphiidae were dominant spider families in all field margins except for RO(ORG). Oxyopidae hold high proportions of the total spiders on rosemary RO(ORG), RO(IPM), and SV (53.85%, 42.86%, and 40%, respectively). In 2022, arachnid communities on RO(IPM) and SV were composed of 11 spider families each, followed by OR, SG, RO(ORG), and WV (10, 9, 8, and 8 arachnid taxa, respectively). Thomisidae and Salticidae had increased their proportions among total spiders in almost all field margins, while Oxyopidae were dominant on all aromatic plants Figure 9B,E).

In 2021, predatory Heteroptera were the most abundant insect group in the samples. Miridae were dominant in almost all field margins (e.g., on OR, 76.67%), and Athocoridae were dominant in SV and SG (63.16% and 47.46%, respectively). In 2022, mirids were superdominant in OR and WV (89.67% and 84.88%, respectively). In 2022, Anthocoridae represent 54.10% of insect predators on SV, and Mantodea reached high proportions on RO(ORG) and RO(IPM) (41.67% and 30%, respectively) while absent in 2021 (Figure 9C,F).

**Figure 10:**
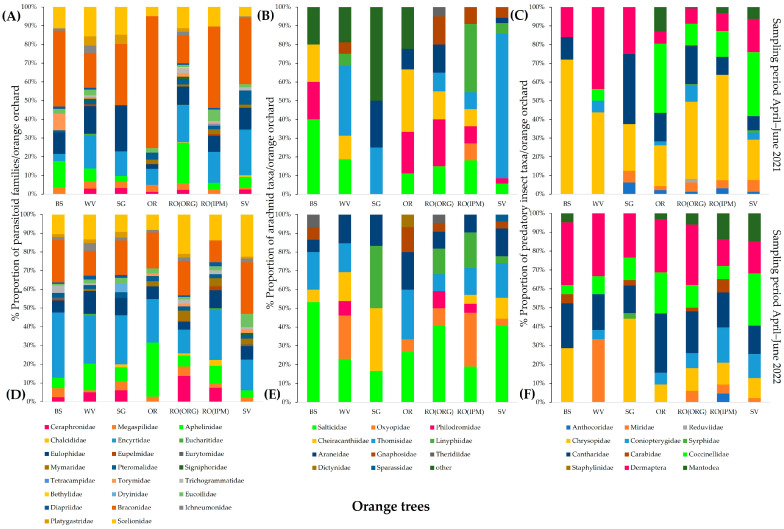
Community composition of natural enemies on orange trees in orchards with different field margin management. Sampling period from April to June 2021: (**A**) Hymenoptera parasitoids; (**B**) arachnid predators; (**C**) insect predators. Sampling period from April to June 2022: (**D**) Hymenoptera parasitoids; (**E**) arachnid predators; (**F**) insect predators. WV—weed vegetation; RO(ORG)—rosemary hedgerow (organic orchard); RO(IPM)—rosemary hedgerow (IPM orchard); OR—oregano hedgerow; SG—sage hedgerow; SV—savory hedgerow.

Regarding the diversity of natural enemies on the orange trees, in 2021 the most diverse community of parasitic Hymenoptera was observed in the RO(ORG) orchard (19 families), followed by the WV field (14 families), and the least diverse orchard was SG (8 families). Braconidae was the dominant family on orange trees in all orchards, especially on OR and BS fields (70.37% and 40.51%, respectively). Encyrtidae, Eulophidae, Aphelinidae, and Scelionidae also had high proportions of total parasitoids in most orchards. In 2022, the greatest diversity of parasitic wasps was observed again in the RO(ORG) orchard (17 families). Encyrtidae and Braconidae were dominant on orange trees in all orchards, and Scelionidae held again high proportions on most fields (Figure 10A,D).

In arachnid predators, Salticidae, Cheiracanthiidae, and Thomisidae were the dominant spider families on trees in most orange orchards in 2021. The next year, Salticidae remained the dominant spider family in all fields, while Thomisidae and Araneidae also had increased proportions (Figure 10B,E).

During 2021, Chrysopidae was the dominant predatory insect group on orange trees in all orchards, while Cantharidae, Coccinellidae, and Dermaptera also had high proportions of total predators. In 2022, mirids were superdominant in OR and WV orchards (89.67% and 84.88%, respectively). Dermaptera and Cantharidae were dominant in all orchards, followed by Chrysopidae (Figure 10C,F).

**Figure 11:**
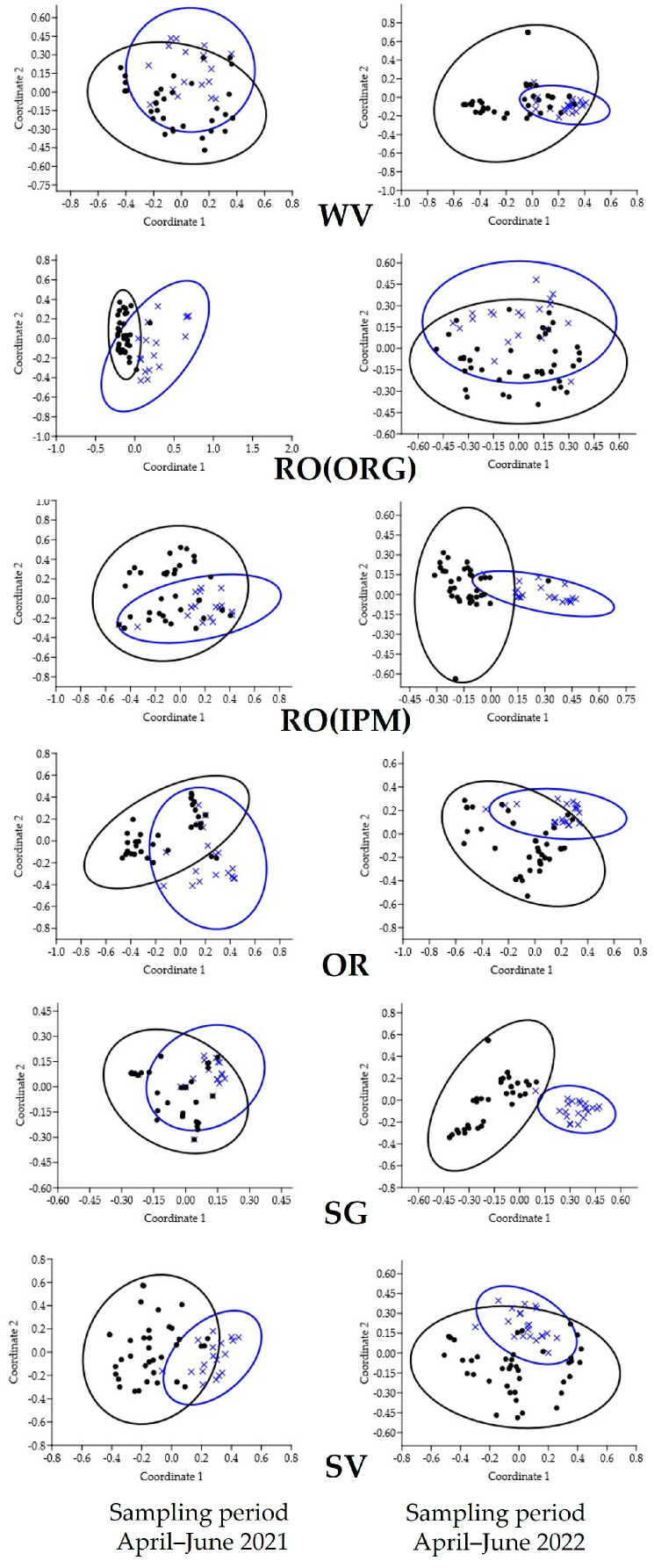
Principle Coordinate Analysis graphs showing the similarity between communities of natural enemies on field margins (blue ellipses) and orange trees (black ellipses) in orchards with different field margin management: WV—weed vegetation; RO(ORG)—rosemary hedgerow (organic orchard); RO(IPM)—rosemary hedgerow (IPM orchard); OR—oregano hedgerow; SG—sage hedgerow; SV—savory hedgerow.

A comparison between the communities of natural enemies on field margins and trees in the same orchard using the PCoA and ANOSIM tests showed greater dissimilarity (higher R values) in 2021. In 2022, lower R values indicate less dissimilar natural enemies’ communities between field margins and trees, while in the SV orchard the communities do not significantly differ (*p* = 0.1738) (Table 1, Figure 11).

#### 3.2.3. Phytophagous Arthropods

Although the impact of hedgerows on arthropod pests of the crop was not one of the aims of this study, we should note that no population outbreak of the common citrus pests or a new pest was recorded on the crop after the establishment of the aromatic plant hedgerows or with respect to the BS and WV orchards. The key pests, according to the farmers’ records and the specimens of phytophagous arthropods found in our suction samples, were aphids, whiteflies, scale insects (mainly *Aonidiela aurantii*), thrips, lepidoptera (mainly *Adoxophyes orana*), and spider mites (mainly *Tetranychus urticae* and *Panonychus citri*), namely in the BS, WV, SG, and RO(IPM) orchards. No insecticides/acaricides were applied in OR and SV orchards, while the farmers applied the standard treatments in the rest of the IPM orchards. Applications in the organic orchard RO(ORG) included *Bacillus thuringiensis* subsp. *kurstaki* and paraffin oil. Regarding other pesticide applications, fungicide treatments (copper hydroxide) in the IPM orchards targeted *Phoma tracheiplila*.

## 4. Discussion

The profile of natural enemies on the aromatic plants oregano, rosemary, sage, and savory, established as hedgerows in the field margins of orange orchards, differed with the plant species. Thus, each species has a different potential as an insectary plant for the conservation of parasitoids, arachnids, and predatory insects that are insect pests, as well as for the relevant abundance and diversity of smaller taxa within these groups. Additionally, differences are evident in the comparison of aromatic plants with the common practice of field margin management of orange orchards, i.e., the maintenance of weed vegetation and bare soil.

Regarding Hymenoptera parasitoids, savory plants attracted more parasitoid wasps compared to weed vegetation and the other aromatic plants (savory > rosemary (ORG) > sage > oregano), with the highest population occurring in late May to June during the flowering period of savory, probably due to the high sugar concentration of its nectar [61].

The attraction of parasitoid wasps to rosemary hedgerows differed in the two orchards, from high in the organic (ORG) field to low in the IPM field (especially in the first sampling period). In any case, the attractance of rosemary to parasitoids cannot be related to flower resources since the plants did not flower during our study. The high parasitoid abundance on rosemary (ORG) hedgerows can be attributed to *Anagrus* spp. (Mymaridae), an egg parasitoid of the Ligurian leafhopper *Eupteryx decemnotata* Rey (Hemiptera: Cicadellidae) [62]. This typhlocybid leafhopper was recorded in high numbers on rosemary and sage hedgerows; however, symptoms of infestation (yellowing) were observed only on rosemary leaves. *E. decemnotata* is an oligophagous species on aromatic plants of the family Lamiaceae and has not been reported to transmit any plant pathogens; therefore, it cannot be harmful for citrus cultivation [63,64].

Even though they had an extended blooming period, sage plants attracted fewer parasitoids than savory, probably due to the difficulty of small parasitic wasps entering the long-tubed flowers of sage [65,66] and/or the low sugar concentration of its nectar [67]. The Ligurian leafhopper abundance did not correspond with an abundance of its parasitoid *Anagrus* spp. on the sage hedgerow as it did in the rosemary hedgerow, an observation that might be worth investigating further for a potential tri-trophic (host plant-herbivore-parasitoid) interaction. Oregano attracted the lowest number of parasitoid individuals, showing a few population peaks only during the flowering period (late May to June), but no data on its nectar attractiveness are available in the literature. In the case of weed vegetation, we consider that low numbers of parasitic Hymenoptera correspond to the poor flowering of the weed species during both sampling periods.

The most abundant parasitoid family in all field margins, on aromatic plants and on weed vegetation, was Scelionidae. The family includes egg parasitoids, which have a wide host range and are efficient biological control agents of pentatomid agricultural pests (Hemiptera: Pentatomidae) [68]. Several other parasitoid families recorded on the field margins include parasitoids, which are efficient biological control agents of citrus pests. The Eulophids *Citrostichus phyllocnistoides* (Narayanan), *Neochrysocharis formosa* (Westwood), and *Pnigalio* spp., which were attracted mostly by savory plants and weeds, are efficient parasitoids of the citrus leafminer *P*. *citrella* [24,69,70]. *Cales noacki* Howard, a parasitoid of the woolly whitefly *A*. *floccosus*, *Aphytis* spp., parasitoids of the California red scale *A*. *aurantii,* and other scale insects, along with other aphelinids, were attracted mostly by savory and sage plants [24]. Savory, sage, and weed vegetation attracted Braconidae, especially Aphidiinae, which are efficient in controlling citrus aphids [71]. Encyrtids, which are also important for biological control of citrus pests such as *P*. *citrella*, scale insects, and the citrus flower moth, *Prays citri* (Lepidoptera: Hyponomeutidae), were present on aromatic plants [24]. As mentioned before, rosemary plants harbored high numbers of Mymaridae, egg parasitoids of insect pests, including Auchenorrhyncha [72].

The orange trees of the organic orchard RO(ORG) had the highest abundance of parasitic Hymenoptera in both years of rosemary hedgerows in the field margins. The communities of parasitoid wasps in all orchards were dominated by the families Encyrtidae, Braconidae, Scelionidae, Aphelinidae, and Eulophidae. Our results are in agreement with similar observations on the community structure of parasitoids in citrus orchards of the Iberian Peninsula [73].

Considering predators, in the first year of the aromatic plant hedgerows in the orchards (2021), weed vegetation harbored significantly more spiders than the aromatic plant hedgerows, while in 2022, the fully grown aromatic plants hosted far more spider individuals. Rosemary had the highest abundance of arachnids, while sage had the lowest. Rosemary shrubs planted around greenhouses have been shown to support a high abundance of spiders [74]. The spider communities consisted mainly of active hunters (e.g., Oxyopidae, Salticidae, Thomisidae, Philodromidae, Cheiracanthiidae, Sparassidae) and secondarily of web-builders (e.g., Araneidae, Theridiidae, Linyphiidae, Tetragnathidae, Uloboridae) [75]. Aromatic plants were colonized mostly by lynx spiders (Oxyopidae), diurnal wandering spiders that prey on a wide variety of insect pests [76]. In contrast, web-builders of the family Linyphiidae dominated on weed vegetation. Crab spiders (Thomisidae) had a strong presence both in field margins and on orange trees. These ambush predators are abundant in orchards and prey on many pests, such as spider mites and aphids [76]. Philodromidae and Theridiidae were collected from both field margins and orange trees, but in small numbers.

On orange trees, arachnids were most abundant in the savory orchard in both sampling periods. The polyphagous jumping spiders (Salticidae) were the dominant group in all orchards. The web-building spider guild was represented mainly by common orb weavers (Araneidae), which are widely abundant in orchards and trap mostly dipteran and homopteran pests on their webs [76]. Several spiders have been reported as predators of citrus pests, including fruit flies, aphids, whiteflies, scale insects, and moths [77,78,79,80]. *Cheiracanthium mildei* Koch (Cheiracanthiidae), a common predator of scale insects and the medfly *C*. *capitata*, was present on aromatic plants and orange trees [81,82]. *Oxyopes heterophthalmus* (Latreille), *O*. *lineatus* Latreille (Oxyopidae), *Agyneta* sp. (Linyphyidae), *Runcinia grammica* (Koch), *Xysticus* sp. (Thomisidae), *Icius hamatus* (Koch), *Heliophanus* spp., *Thyene imperialis* (Rossi) (Salticidae), *Mangora acalypha* (Walckenaer) (Araneidae), and *Olios argelasius* (Walckenaer) (Sparassidae) are some spider taxa collected during our study which are commonly found in citrus and other fruit orchards [83,84,85,86]. Harvestmen (Opiliones) were found only in field margins and in low numbers.

The abundance and diversity profile of insect predators on the aromatic plants/weed vegetation, and orange trees in the respective orchards differed from that of the arachnids. Predatory insects were most abundant on oregano and sage than on the other aromatic plant species and weeds, while the lowest abundance was observed on rosemary. Oregano, sage, and weeds attracted mostly zoophytophagous Mirids such as *Macrolophus* spp., which are efficient biological control agents of soft-bodied pests [87]. Sage and savory hedgerows attracted mainly pirate bugs, *Orius* spp. (Anthocoridae), which are generalist predators very effective in thrips control [88]. The attraction of *Orius* spp. to aromatic flowering plants of the Lamiaceae family has also been reported in other studies [89].

Coccinellid predators, including the specific predator *Icerya purchasi* Maskell (Hemiptera: Monophlebidae), *Rodolia cardinalis* (Mulsant), and the generalist predators of aphids and scale insects, *Scymnus* spp., were present on sage and rosemary hedgerows, although in low numbers [73]. Elekcioğlu [90] reported a great number of ladybug species (Coccinellidae) found on aromatic and medicinal plants of the Lamiaceae family. Sage and rosemary hedgerows in our study also hosted lacewings (Chrysopidae), earwigs (Dermaptera), and soldier beetles (Cantharidae). Lacewings are polyphagous predators and important biological control agents for aphids and other soft-bodied phytophagous insects [91]. In the second sampling period (2022), the fully grown rosemary plants hosted the European mantid, *Mantis religiosa* L. (Mantodea: Mantidae), a large solitary predator that prefers woody plants as roosting substrate [92].

Predatory insects in both sampling periods were most abundant on orange trees in the RO(ORG), RO(IPM), and SV orchards. In all orchards, the communities of predaceous insects consisted mainly of Neuroptera (mainly Chrysopidae), Dermaptera, and Coleoptera. Omnivorous species, such as the European earwig *Forficula auricularia* L. (Dermaptera: Forficulidae) and the red soldier beetle *Rhagonycha fulva* (Scopoli) (Coleoptera: Cantharidae), which represented high proportions of the samples, are well known predators of aphids and other small insects [93,94]. Coccinellid predators were found on orange trees in all study orchards. Mantodea were also present mostly in the rosemary RO(ORG), RO(IPM), and savory (SV) orchard. The neuropteran predator, *Conwentzia psociformis* (Curtis) (Coniopterygidae), that was present on orange trees of all orchards except for those with field margins of bare soil (BS) and sage (SG), and it was not found in the field margins, preys on citrus aphids, scale insects, and spider mites [95,96].

Natural enemy communities showed changes over time since the first year of the hedgerows’ establishment, probably due to the different ways of dispersal of the different functional groups. We assume that walking arthropods such as spiders or large predators such as Mantodea must have taken longer to colonize the hedgerows than the flying ones (Hymenoptera parasitoids). Thus, their abundance increased in the second year of the hedgerows’ presence in the orange fields. Moreover, we consider that the movement of natural enemies between the orange trees and the field margins has led to an increase in the similarity of these communities in the second year. Moreover, the impact of the aromatic plant hedgerows on conservation of natural enemies of pests in relation to the weed flora in the field margins in the baseline year varied with the plant species and the natural enemy group, i.e., sage plants favored predatory insects but harbored low populations of Hymenoptera parasitoids compared to the main flowering weed species, *M*. *chamomilla* and *R*. *raphanistrum*; savory plants had a positive effect on the abundance of parasitoids and arachnid predators; rosemary hedgerows served as a most favorable habitat for spiders in comparison to the pre-existing weed cover. It is worth mentioning that no adverse effects, such as outbreaks of common citrus pests or the introduction of a new pest, were recorded on the crop after the establishment of the aromatic plant hedgerows. Overall, our findings support the implementation of aromatic plants in conservation practices for natural enemies of arthropod pests in citrus orchards and possibly incorporating suitable wild flowering species of the weed flora. Further research should focus on the delivery of biological control services from the aromatic plant hedgerows to the crop.

## 5. Conclusions

The present study examined the potential of hedgerows from Mediterranean aromatic plant species, i.e., oregano, rosemary, sage, and savory, in orange field margins to function as reservoirs of natural enemies of citrus pests in comparison to the common management practice of bare soil or weed vegetation. The impact of the aromatic plant hedgerows on the conservation of parasitoid wasps, spiders, and predators of insect pests varied with the aromatic plant species and natural enemy group. Savory attracted more parasitoids compared to weed vegetation and the other aromatic plants (savory > organic rosemary > sage > oregano). Weed vegetation hosted more arachnid predators than the aromatic plant hedgerows in their first year in the orchard, but this was reversed with the fully grown plants in the following year (most abundant on rosemary). Oregano and sage favor insect predators. The similarity of natural enemy communities on the field margins and on the orange trees seems to increase with time, indicating the insects’ movement between the field margins and the trees. The results support the use of the tested aromatic plant species in conservation practices for targeted groups of beneficial arthropods in orange orchards, considering also the possible incorporation of suitable wild flowering plants from the weed flora.

## Figures and Tables

**Table 1:** One-way ANOSIM between communities of natural enemies on field margins and orange trees in orchards with different field margin management. WV—weed vegetation; RO(ORG)—rosemary hedgerow (organic orchard); RO(IPM)—rosemary hedgerow (IPM orchard); OR—oregano hedgerow; SG—sage hedgerow; SV—savory hedgerow.

	Sampling Period April–June 2021		Sampling Period April–June 2022	
Field	*R*	*p*	*R*	*p*
WV	0.1372	0.0056	0.157	0.001
RO(ORG)	0.5026	0.0001	0.1447	0.0067
RO(IPM)	0.2631	0.0001	0.1793	0.0001
OR	0.2021	0.0005	0.1328	0.0003
SG	0.0736	0.0499	0.1344	0.0027
SV	0.3154	0.0001	0.0393	0.1738

## Data Availability

The data presented in this study are available upon request from the corresponding authors.

## Supplementary Materials

The following supporting information can be downloaded at: https://www.mdpi.com/article/10.3390/insects14040391/s1, Table S1: Plant protection interventions in the orange orchards (BS, WV, SG, OR, SV, RO(ORG), and RO(IPM)) during the study; Table S2: Natural enemies (taxa) recorded in the field margins (FM) and on orange trees (OT) of the experimental orchards (BS, WV, SG, OR, SV, RO(ORG), and RO(IPM)) during the study; Table S3: Mean number (±SE) of natural enemies (parasitoids, arachnid predators, and insect predators) in field margins in four orange orchards (WV, SG, SV, and RO(IPM)) at the baseline year 2020 (field margin management using the usual farmers’ practice); Table S4: Mean number (±SE) of natural enemies (parasitoids, arachnid predators, and insect predators) on orange trees in seven orchards (BS, WV, SG, OR, SV, RO(ORG), and RO(IPM)) at the baseline year 2020 (field margin management using the usual farmers’ practice); Table S5: Mean number (±SE) of natural enemies (parasitoids, arachnid predators, and insect predators) in field margins in orange orchards with different field margin management during April–June 2021; Table S6: Mean number (±SE) of natural enemies (parasitoids, arachnid predators, and insect predators) on orange trees in orchards with different field margin management during April–June 2021; Table S7: Mean number (±SE) of natural enemies (parasitoids, arachnid predators, and insect predators) in field margins in orange orchards with different field margin management during April–June 2022; Table S8: Mean number (±SE) of natural enemies (parasitoids, arachnid predators, and insect predators) on orange trees in orchards with different field margin management during April–June 2022.
