# Supplementary material for: Potential of Hedgerows with Aromatic Plants as Reservoirs of Natural Enemies of Pests in Orange Orchards
Source: Insects. 2023 Apr 17;14(4):391. doi: 10.3390/insects14040391 (PMC10141982; doi:10.3390/insects14040391)
Supplement: Supplementary file 1 [file insects-14-00391-s001.zip › insects-2291353-supplementary.pdf]

Table S1. Plant protection interventions in the orange orchards (BS, WV, SG, OR, SV, RO(ORG), RO(IPM)) during the study.

Field	Target pest /weeds	Chemical control - active substances
BS	aphids, whiteflies, scale insects, thrips, lepidoptera, spider mites	chlorpyrifos, acetamiprid, tau-fluvalinate, hexythiazox, abamectin, phosmet, acetamiprid, pyriproxyfen, sulfoxaflor, paraffin oil
	Phoma tracheiphila	copper hydroxide
	various weeds	glyphosate, penoxsulam, florasulam, flazasulfuron
WV	aphids, whiteflies, scale insects, thrips, spider mites	spirodiclofen, spirotetramat, pyriproxyfen, paraffin oil
	Phoma tracheiphila	copper hydroxide
	various weeds	glyphosate
SG	aphids, whiteflies, scale insects, thrips, lepidoptera, medfly, spider mites	deltamethrin, spirotetramat, paraffin oil, pyriproxyfen, abamectin, tau-fluvalinate
	Phoma tracheiphila	copper hydroxide
	various weeds	glyphosate
OR	*Phoma tracheiphila*	copper hydroxide
	various weeds	glyphosate
SV	*Phoma tracheiphila*	copper hydroxide
	various weeds	glyphosate
RO(ORG)	aphids, whiteflies, scale insects, lepidoptera, spider mites	*Bacillus thuringiensis* subsp. *kurstaki*, paraffin oil
	Phoma tracheiphila	copper hydroxide
RO(IPM)	aphids, whiteflies, scale insects, thrips, spider mites	abamectin, phosmet
	Phoma tracheiphila	copper hydroxide
	various weeds	glyphosate

BS: bare soil; WV: weed vegetation; SG: sage hedgerow; OR: oregano hedgerow; SV: savory hedgerow; RO(ORG): rosemary hedgerow (organic orchard); RO(IPM): rosemary hedgerow (IPM orchard)

Table S2. Natural enemies (taxa) recorded in the field margins (FM) and on orange trees (OT) of the experimental orchards (BS, WV, SG, OR, SV, RO(ORG), RO(IPM)) during the study.

		BS	WV		SG		OR		SV		RO (ORG)		RO (IPM)	
		OT	FM	OT	FM	OT	FM	OT	FM	OT	FM	OT	FM	OT
**Hymenoptera parasitoids**														
Ceraphronoidea	Ceraphronidae	+	+	+	+	+		+	+	+	+	+	+	+
	Megaspilidae	+	+	+	+	+	+	+	+	+	+	+	+	+
Chalcidoidea	Aphelinidae	+	+	+	+	+	+	+	+	+	+	+	+	+
	Chalcididae		+		+	+	+			+	+	+	+	+
	Encyrtidae	+	+	+	+	+	+	+	+	+	+	+	+	+
	Eucharitidae		+	+								+		+
	Eulophidae	+	+	+	+	+	+	+	+	+	+	+	+	+
	Eupelmidae	+	+	+			+					+	+	+
	Eurytomidae	+							+	+				
	Mymaridae		+	+	+	+		+	+	+	+	+	+	+
	Perilampidae		+		+		+		+					
	Pteromalidae	+	+	+	+	+	+	+	+	+	+	+	+	+
	Signiphoridae			+			+		+	+		+		
	Tetracampidae		+		+				+					+
	Torymidae	+	+	+	+			+	+	+		+	+	+
	Trichogrammatidae	+	+	+	+	+	+	+	+	+	+	+		+
Chrysidoidea	Bethylidae	+			+		+	+						
	Dryinidae				+	+			+		+			
Cynipoidea	Eucoilidae	+	+	+	+	+	+	+	+	+		+	+	+
Diaprodoidea	Diapriidae	+	+	+	+			+	+			+		+
Ichneumonoidea	Braconidae	+	+	+	+	+	+	+	+	+	+	+	+	+
	Ichneumonidae	+	+	+	+	+	+	+	+	+		+	+	
Platygastroidea	Platygastridae	+	+	+		+				+	+	+		+
	Scelionidae	+	+	+	+	+	+	+	+	+	+	+	+	+
Trigonaloidea	Trigonalidae				+									
**Arachnid predators**														
Araneae (*spiders*)	Araneidae (*orb weavers*)	+	+	+	+	+	+	+	+		+	+	+	+
	Cheiracanthiidae (*yellow sac spiders*)	+		+	+	+	+	+	+	+	+	+	+	+
	Dictynidae							+						
	Gnaphosidae	+	+	+				+			+	+		+
	Linyphiidae (*money spiders*)		+	+	+	+	+	+	+	+	+	+	+	+
	Lycosidae (*wolf spiders*)		+				+						+	
	Oxyopidae (*lynx spiders*)	+	+	+	+		+	+	+	+	+		+	+
	Philodromidae	+	+	+	+		+	+	+	+	+	+	+	+
	Pisauridae						+		+		+		+	
	Salticidae (*jumping spiders*)	+	+	+	+	+	+	+	+	+	+	+		+
	Sparassidae									+				
	Tetragnathidae												+	
	Theridiidae (*cobweb spiders*)	+	+		+		+		+			+	+	
	Thomisidae (*crab spiders*)	+	+	+	+	+	+	+	+	+	+	+	+	+
	Uloboridae		+						+					
	Zodariidae		+											

	Other (unidentified immatures)	+	+	+		+	+	+	+	+	+	+	+	
Opiliones (*harvestmen*)			+		+		+		+		+		+	
**Insect predators**														
Coleoptera	Cantharidae (*soldier beetles*)	+		+	+	+	+	+	+	+	+	+		+
	Carabidae (*ground beetles*)	+	+			+	+					+		+
	Coccinellidae (*ladybirds*)	+	+	+	+	+	+	+	+	+	+	+	+	+
	Staphylinidae (*rove beetles*)		+		+				+		+			
Dermaptera (*earwigs*)		+		+	+	+	+	+	+	+	+	+	+	+
Diptera	Syrphidae (*hoverflies*)		+	+	+	+	+		+	+	+	+	+	
Hemiptera	Anthocoridae (*pirate bugs*)		+		+	+	+	+	+	+	+	+	+	+
	Miridae (*mirid bugs*)		+	+	+	+	+	+	+	+	+	+		+
	Reduviidae (*assassin bugs*)		+		+		+		+		+	+	+	
Mantodea (*mantids*)		+	+		+		+	+	+	+	+	+	+	+
Neuroptera	Chrysopidae (*lacewings*)	+	+	+	+	+	+	+	+	+	+	+	+	+
	Coniopterygidae			+				+		+		+		+

BS: bare soil; WV: weed vegetation; SG: sage hedgerow; OR: oregano hedgerow; SV: savory hedgerow; RO(ORG): rosemary hedgerow (organic orchard); RO(IPM): rosemary hedgerow (IPM orchard)

Table S3. Mean number (±SE) of natural enemies (parasitoids, arachnid predators, insect predators) in field margins in four orange orchards (WV, SG, SV, RO(IPM)) at the baseline year 2020 (field margin management using the usual farmers' practice).

**Hymenoptera parasitoids**		
**Field margin**	**28-29/4**	**5-6/5**
**WV**	7.7±3.2 A	7.7±2.7 B
**SG**	32.7±10.2 A	38.0±8.7 A
**SV**	12.0±3.5 A	9.3±3.5 AB
**RO(IPM)**	5.7±2.3 A	9.7±2.2 AB
**2-way ANOVA** Field: $F_{3,16}$= 8.7735, P = 0.0011; Date: $F_{1,16}$ = 0.0434, P = 0.8375; Field*Date: $F_{3,16}$ = 1.2161, P = 0.336		
**Arachnid predators**		
**WV**	8.0±3.1 A	5.3±4.3 A
**SG**	1.0±0.6 B	1.0±1.0 A
**SV**	0.3±0.3 B	1.0±0.6 A
**RO(IPM)**	2.0±0.6 AB	2.0±0.0 A
**2-way ANOVA** Field: $F_{3,16}$= 4.15, P = 0.0236; Date: $F_{1,16}$ = 2.7334, P = 0.1178; Field*Date: $F_{3,16}$ = 4.6373, P = 0.0162		
**Insect predators**		
**WV**	2.3±0.7 AB	2.0±0.6 A
**SG**	5.0±0.6 A	7.7±3.8 A
**SV**	1.3±0.3 B	1.3±0.9 A
**RO(IPM)**	2.3±0.7 AB	0.3±0.3 A
**2-way ANOVA** Field: $F_{3,16}$= 5.487, P = 0.0087; Date: $F_{1,16}$ = 2.8799, P = 0.1091; Field*Date: $F_{3,16}$ = 0.9842, P = 0.425		

WV: weed vegetation; RO(IPM): rosemary hedgerow (IPM orchard); OR: oregano hedgerow; SG: sage hedgerow; SV: savory hedgerow. Capital letters indicate significant differences between orchards.

Table S4. Mean number (±SE) of natural enemies (parasitoids, arachnid predators, insect predators) on orange trees in seven orchards (BS, WV, SG, OR, SV, RO(ORG), RO(IPM)) at the baseline year 2020 (field margin management using the usual farmers' practice).

	**Hymenoptera parasitoids**	
**Orange trees**	**18-25/6**	**20-21/7**
**BS**	4.3±1.9 Aa	8.5±3.6 ABCa
**WV**	2.0±0.7 Aa	1.5±0.7 Ca
**SG**	8.2±2.0 Aa	6.7±2.4 ABCa
**OR**	6.5±1.4 Ab	18.0±3.4 Aa
**SV**	5.7±1.4 Aa	14.2±4.6 ABa
**RO(ORG)**	8.5±2.2 Aa	4.7±2.0 BCa
**RO(IPM)**	5.3±1.2 Aa	8.3±1.6 ABa
**2-way ANOVA** Field: $F_{6,70} = 3.8497$, $P = 0.0022$; Date: $F_{1,70} = 5.8961$, $P = 0.0177$; Field*Date: $F_{6,70} = 2.7665$, $P = 0.018$		
	**Arachnid predators**	
**BS**	0.0±0.0 Ba	0.7±0.5 BCa
**WV**	0.3±0.2 Ba	0.7±0.2 BCa
**SG**	0.3±0.2 Ba	0.2±0.2 Ca
**OR**	3.2±0.6 Aa	6.2±1.6 Aa
**SV**	0.8±0.5 Ba	2.0±0.4 ABa
**RO(ORG)**	0.8±0.4 Ba	1.2±0.4 BCa
**RO(IPM)**	0.8±0.5 Ba	1.8±0.7 ABCa
**2-way ANOVA** Field: $F_{6,70} = 13.4819$, $P < 0.0001$; Date: $F_{1,70} = 9.5304$, $P = 0.0029$; Field*Date: $F_{6,70} = 0.8211$, $P = 0.5574$		
	**Insect predators**	
**BS**	0.0±0.0 A	0.3±0.2 B
**WV**	0.5±0.3 A	0.5±0.2 B
**SG**	0.7±0.3 A	0.2±0.2 B
**OR**	2.2±0.9 A	3.2±0.2 A
**SV**	1.5±0.6 A	1.0±0.4 B
**RO(ORG)**	2.0±1.2 A	1.3±0.6 AB
**RO(IPM)**	0.7±0.3 A	0.8±0.5 B
**2-way ANOVA** Field: $F_{6,70} = 5.7513$, $P < 0.0001$; Date: $F_{1,70} = 0.0823$, $P = 0.775$; Field*Date: $F_{6,70} = 0.7871$, $P = 0.583$		

BS: bare soil; WV: weed vegetation; SG: sage hedgerow; OR: oregano hedgerow; SV: savory hedgerow. RO(ORG): rosemary hedgerow (organic orchard); RO(IPM): rosemary hedgerow (IPM orchard). Capital letters indicate significant differences between orchards while small letters indicate significant differences between assessment dates.

Table S5. Mean number (±SE) of natural enemies (parasitoids, arachnid predators, insect predators) in field margins in orange orchards with different field margin management during April-June 2021.

Field margin	28/4	5-7/5	12-13/5	21/5	27/5	7/6
**Hymenoptera parasitoids**						
**WV**	2.3±1.2 ABa	7.0±3.5 ABa	3.7±0.9 ABa	5.3±2.3 ABa	4.3±0.9 BCa	4.7±2.3 BCa
**SG**	1.0±0.6 Bc	2.7±1.2 ABbc	8.0±1.5 ABab	6.7±0.3 Aab	6.7±0.9 ABCab	16.7±2.3 ABa
**OR**	3.0±1.2 ABab	0.7±0.7 Bb	2.0±1.2 ABab	3.0±1.0 ABab	14.0±8.0 ABCa	5.7±1.3 ABCab
**SV**	11.0±3.8 Aab	12.3±3.7 Aa	12.0±3.5 Aa	1.3±0.9 Bb	20.3±5.8 Aa	37.7±19.7 Aa
**RO(ORG)**	0.3±0.3 Bb	21.3±9.6 Aa	1.3±0.9 ABb	2.7±0.3 ABab	14.7±1.9 ABa	5.3±2.0 BCab
**RO(IPM)**	1.7±0.3 ABa	0.3±0.3Ba	1.7±1.7 Ba	3.0±1.2 ABa	2.7±0.7 Ca	1.3±0.3 Ca
**2-way ANOVA** Field: $F_{5,72}$= 13.8154, $P < 0.0001$; Date: $F_{5,72}$ = 9.6705, $P < 0.0001$; Field*Date: $F_{25,72}$ = 4.0936, $P < 0.0001$						
**Arachnid predators**						
**WV**	3.0±0.6 A	2.0±1.0 A	11.0±2.9 A	5.3±2.7 A	3.7±2.2 A	6.0±1.7 A
**SG**	0.0±0.0 C	0.3±0.3 A	0.0±0.0 C	0.3±0.3 A	1.0±0.6 A	0.3±0.3 B
**OR**	0.3±0.3 BC	0.3±0.3 A	1.0±0.6 BC	0.7±0.3 A	0.7±0.3 A	0.7±0.3 B
**SV**	3.3±1.3 A	3.0±0.6 A	3.0±0.6 AB	2.0±1.0 A	2.0±1.0 A	6.7±3.3 A
**RO(ORG)**	4.7±0.3 A	3.3±0.9 A	1.7±0.9 BC	0.3±0.3 A	1.7±0.9 A	1.3±0.3 AB
**RO(IPM)**	2.0±0.6 AB	1.3±0.9 A	0.0±0.0 C	1.0±0.0 A	1.7±0.7 A	1.0±0.6 AB
**2-way ANOVA** Field: $F_{5,72}$= 19.4261, $P < 0.0001$; Date: $F_{5,72}$ = 0.811, $P = 0.5457$; Field*Date: $F_{25,72}$ = 1.8213, $P = 0.0257$						
**Insect predators**						
**WV**	1.3±0.9 Aa	0.7±0.7 Aa	2.0±0.6 ABa	6.0±1.7 Aa	2.0±1.5 Aa	2.3±0.9 ABa
**SG**	1.0±0.6 Abc	0.3±0.3 Ac	4.7±0.3 Aab	7.0±1.7 Aa	4.0±1.7 Aabc	2.7±0.7 ABabc
**OR**	1.3±0.9 Aa	2.7±0.3 Aa	1.3±0.7 ABCa	5.7±1.9 Aa	8.7±3.2 Aa	6.0±1.7 Aa
**SV**	1.0±0.6 Aa	1.7±0.9 Aa	0.3±0.3 BCa	1.3±0.9 Aa	3.3±1.7 Aa	5.0±2.1 ABa
**RO(ORG)**	0.0±0.0 Aa	0.7±0.3 Aa	0.7±0.3 BCa	1.7±1.2 Aa	1.7±1.7 Aa	1.7±0.3 ABa
**RO(IPM)**	0.0±0.0 Aa	0.3±0.3 Aa	0.0±0.0 Ca	0.7±0.3 Aa	0.3±0.3 Aa	1.0±0.6 Ba
**2-way ANOVA** Field: $F_{5,72}$= 10.6998, $P < 0.0001$; Date: $F_{5,72}$ = 7.6176, $P < 0.0001$; Field*Date: $F_{25,72}$ = 1.4124, $P = 0.13$						

WV: weed vegetation; RO(ORG): rosemary hedgerow (organic orchard); RO(IPM): rosemary hedgerow (IPM orchard); OR: oregano hedgerow; SG: sage hedgerow; SV: savory hedgerow. Capital letters indicate significant differences between orchards while small letters indicate significant differences between assessment dates.

Table S6. Mean number (±SE) of natural enemies (parasitoids, arachnid predators, insect predators) on orange trees in orchards with different field margin management during April-June 2021.

Orange trees	28/4	5-7/5	12-13/5	21/5	27/5	7/6
**Hymenoptera parasitoids**						
**BS**	3.8±1.0 ABa	0.7±0.3 Ba	4.5±1.5 ABa	1.0±0.5 Da	0.7±0.3 Ca	2.5±1.7 BCa
**WV**	3.7±1.0 ABa	1.0±0.4 Ba	4.0±1.1 ABa	1.7±0.7 Da	4.0±1.0 ABa	2.7±1.1 ABCa
**SG**	1.3±0.6 Ba	0.8±0.3 Ba	2.2±0.3 ABa	2.7±0.3 BCDa	2.2±0.7 BCa	1.0±0.4 Ca
**OR**	1.7±0.7 Bbc	1.5±0.5 Bbc	0.7±0.3 Bc	6.2±1.3 ABCa	2.7±0.6 BCab	0.8±0.2 Cbc
**SV**	2.0±0.5 Ba	3.0±1.5 Ba	2.7±0.9 ABa	2.5±1.0 CDa	3.3±0.7 Ba	6.3±1.6 ABa
**RO(ORG)**	7.2±1.2 Aa	13.5±1.8 Aa	6.3±0.9 Aa	10.5±1.9 Aa	10.5±1.7 Aa	9.8±2.4 Aa
**RO(IPM)**	2.2±0.6 ABb	1.7±0.7 Bb	1.5±0.6 Bb	8.3±1.7 ABa	4.2±1.5 Bab	1.3±0.6 BCb
**2-way ANOVA** Field: $F_{6,210}$ = 26.2251, P < 0.0001; Date: $F_{5,210}$ = 3.4245, P = 0.0054; Field*Date: $F_{30,210}$ =3.2675, P < 0.0001						
**Arachnid predators**						
**BS**	0.2±0.2 A	0.0±0.0 A	0.3±0.2 A	0.0±0.0 A	0.2±0.2 A	0.2±0.2 AB
**WV**	0.5±0.2 A	0.2±0.2 A	0.7±0.7 A	0.2±0.2 A	1.0±0.4 A	0.2±0.2 AB
**SG**	0.0±0.0 A	0.2±0.2 A	0.5±0.3 A	0.0±0.0 A	0.0±0.0 A	0.0±0.0B AB
**OR**	0.3±0.2 A	0.0±0.0 A	0.2±0.2 A	0.0±0.0 A	0.2±0.2 A	0.8±0.4 AB
**SV**	0.3±0.2 A	0.2±0.2 A	0.3±0.2 A	0.5±0.3 A	0.2±0.2 A	4.3±3.5 A
**RO(ORG)**	1.0±0.5 A	0.5±0.2 A	0.2±0.2 A	0.7±0.5 A	0.8±0.5 A	0.2±0.2 AB
**RO(IPM)**	0.2±0.2 A	0.2±0.2 A	0.3±0.2 A	0.5±0.2 A	0.7±0.3 A	0.0±0.0 B
**2-way ANOVA** Field: $F_{6,210}$ = 2.3676, P < 0.0001; Date: $F_{5,210}$ = 0.8307, P = 0.5291; Field*Date: $F_{30,210}$ = 1.4285, P = 0.0785						
**Insect predators**						
**BS**	0.2±0.2 B	0.5±0.2 BC	0.8±0.5 B	0.3±0.2 B	0.7±0.3 B	1.7±0.9 AB
**WV**	0.0±0.0 B	0.5±0.5 C	0.2±0.2 B	0.3±0.2 B	1.3±0.8 B	0.3±0.2 B
**SG**	0.3±0.3 B	0.2±0.2 C	0.8±0.5 B	0.7±0.2 AB	0.2±0.2 B	0.5±0.3 B
**OR**	0.8±0.3 B	0.8±0.4 BC	0.5±0.2 B	2.7±1.7 AB	1.8±0.8 AB	1.0±0.5 AB
**SV**	1.0±0.4 AB	2.5±0.6 AB	2.7±1.4 AB	1.5±0.5 AB	1.8±0.5 AB	3.7±1.0 A
**RO(ORG)**	4.5±1.4 A	4.3±0.8 A	5.5±1.8 A	3.7±1.2 AB	4.7±1.0 A	3.7±0.8 A
**RO(IPM)**	1.0±0.3 AB	0.7±0.2 BC	1.0±0.3 B	4.5±1.5 A	4.8±1.0 A	3.7±0.8 A
**2-way ANOVA** Field: $F_{6,210}$ = 29.5878, P = 0.0311; Date: $F_{5,210}$ = 3.4502, P = 0.0051; Field*Date: $F_{30,210}$ = 1.3416, P = 0.121						

BS: bare soil; WV: weed vegetation; SG: sage hedgerow; OR: oregano hedgerow; SV: savory hedgerow; RO(ORG): rosemary hedgerow (organic orchard); RO(IPM): rosemary hedgerow (IPM orchard). Capital letters indicate significant differences between orchards while small letters indicate significant differences between assessment dates.

Table S7. Mean number (±SE) of natural enemies (parasitoids, arachnid predators, insect predators) in field margins in orange orchards with different field margin management during April-June 2022.

**Field margin**	**26-27/4**	**5/5**	**12-13/5**	**21/5**	**27/5**	**7/6**	**23-24/6**
**Hymenoptera parasitoids**							
**WV**	4.3±2.4 ABC	4.3±2.4 A	4.0±1.0 A	2.7±0.9 BC	3.3±1.8 BC	1.7±0.9 B	3.7±1.2 BC
**SG**	10.0±2.9 A	8.0±2.9 A	7.3±2.6 A	1.3±0.7 C	1.3±0.9 BC	6.0±2.5 AB	5.3±1.2 B
**OR**	1.0±0.6 BC	0.3±0.3 A	1.7±0.7 A	5.7±2.7 BC	5.3±1.3 ABC	32.7±12.4 A	4.0±1.5 BC
**SV**	6.3±2.4 AB	8.7±2.4 A	11.7±2.6 A	10.3±1.2 AB	15.7±5.6 AB	21.7±11.7 AB	34.3±7.5 A
**RO(ORG)**	3.0±0.6 ABC	3.3±1.2 A	2.7±1.8 A	3.7±1.3 BC	41.0±20.4 A	5.0±1.7 AB	5.3±0.3 B
**RO(IPM)**	0.3±0.3 C	2.3±2.3 A	8.7±7.2 A	34.7±9.4 A	0.0±0.0 C	3.0±2.5 B	1.0±1.0 C
**2-way ANOVA** Field: $F_{5,84}$= 12.2624, $P < 0.0001$; Date: $F_{6,84}$ = 2.1135, $P = 0.0601$; Field*Date: $F_{30,84}$ = 4.4539, $P < 0.0001$							
**Arachnid predators**							
**WV**	0.3±0.3 Ba	4.3±1.2 Aa	2.7±2.2 Aa	2.0±1.2 ABa	1.7±0.7 Aa	4.3±1.7 ABa	5.3±1.2 BCa
**SG**	0.3±0.3 Ba	2.3±0.9 ABa	2.0±1.0 Aa	0.0±0.0 Ba	0.3±0.3 Aa	1.3±0.7 Ba	1.7±1.2 Ca
**OR**	1.7±0.3 ABbc	0.0±0.0 Bc	2.3±0.3 Abc	1.7±0.9 ABbc	0.7±0.7 Ac	7.3±2.2 ABab	17.7±2.7 ABa
**SV**	2.3±0.7 ABabc	0.0±0.0 Bc	4.0±2.1 Aabc	1.0±1.0 ABbc	11.3±7.5 Aab	10.3±0.3 Aa	17.3±4.3 ABa
**RO(ORG)**	2.7±1.5 ABb	2.0±1.0 ABb	0.3±0.3 Ab	2.3±0.3 ABb	1.3±0.9 Ab	3.3±0.7 ABab	16.3±4.1 ABa
**RO(IPM)**	7.7±3.7 Ab	7.7±3.7 Ab	4.0±0.6 Ab	5.7±0.9 Ab	4.0±1.5 Ab	11.0±1.0 Aab	28.7±6.3 Aa
**2-way ANOVA** Field: $F_{5,84}$= 16.7319, $P < 0.0001$; Date: $F_{6,84}$ = 19.4021, $P < 0.0001$; Field*Date: $F_{30,84}$ = 2.8298, $P = 0.0001$							
**Insect predators**							
**WV**	2.3±2.3 Ba	4.3±1.2 BCa	6.3±1.5 Aa	1.7±0.7 Aa	3.7±1.3 Ba	4.7±2.3 ABa	5.7±3.7 Aa
**SG**	5.0±0.6 ABa	10.0±2.5 ABa	6.0±2.0 ABa	6.3±3.2 Aa	11.7±1.2 Aa	11.0±2.6 Aa	4.0±0.6 Aa
**OR**	31.0±13.5 Aa	18.0±4.0 Aa	1.3±0.9 BCb	3.3±1.3 Aab	2.7±0.3 Bab	5.7±0.7 ABab	1.0±0.6 Ab
**SV**	1.0±0.0 Babc	0.7±0.7 CDbc	0.0±0.0 Cc	1.7±0.9 Aabc	3.3±0.9 Babc	5.7±1.9 ABab	8.3±4.8 Aa
**RO(ORG)**	2.3±0.3 ABa	0.3±0.3 Da	0.7±0.7 Ca	2.0±1.5 Aa	1.0±0.6 BCa	2.0±1.0 ABa	0.3±0.3 Aa
**RO(IPM)**	0.0±0.0 Ba	0.3±0.3 Da	0.0±0.0 Ca	0.3±0.3 Aa	0.0±0.0 Ca	1.3±0.9 Ba	1.7±1.2 Aa
**2-way ANOVA** Field: $F_{5,84}$= 27.4573, $P < 0.0001$; Date: $F_{6,84}$ = 2.9589, $P = 0.0114$; Field*Date: $F_{30,84}$ = 3.192. $P < 0.0001$							

WV: weed vegetation; RO(ORG): rosemary hedgerow (organic orchard); RO(IPM): rosemary hedgerow (IPM orchard); OR: oregano hedgerow; SG: sage hedgerow; SV: savory hedgerow. Capital letters indicate significant differences between orchards while small letters indicate significant differences between assessment dates.

Table S8. Mean number (±SE) of natural enemies (parasitoids, arachnid predators, insect predators) on orange trees in orchards with different field margin management during April-June 2022.

**Orange trees**	**26-27/4**	**5/5**	**12-13/5**	**21/5**	**27/5**	**7/6**	**23-24/6**
**Hymenoptera parasitoids**							
**BS**	0.5±0.2 Ac	0.5±0.3 Bc	1.2±0.5 Bbc	5.7±2.9 Aab	2.2±0.8 BCbc	3.0±0.8 ABabc	7.7±1.3 Aa
**WV**	0.3±0.3 Ac	1.3±0.3 ABbc	3.0±0.7 ABab	3.0±0.7 Aab	1.8±0.7 BCbc	1.5±0.4 ABbc	5.3±1.1 ABa
**SG**	0.8±0.3 Aab	1.3±0.4 ABab	1.2±0.5 Bab	2.0±0.6 Aab	0.5±0.3 Cb	1.7±0.8 ABab	3.3±0.6 ABa
**OR**	0.8±0.5 Ab	1.3±0.8 ABb	1.0±0.4 Bb	1.5±0.5 Aab	0.8±0.4 BCb	0.3±0.2 Bb	6.3±1.5 ABa
**SV**	1.7±0.3 Aa	2.7±1.3 ABa	1.0±0.3 Ba	2.5±0.6 Aa	3.3±0.8 ABa	2.2±0.9 ABa	3.0±0.5 ABa
**RO(ORG)**	1.0±0.5 Ab	6.7±2.4 Aa	6.7±1.6 Aa	4.7±1.1 Aa	8.5±1.5 Aa	4.8±1.1 Aa	4.0±0.5 ABa
**RO(IPM)**	0.8±0.5 Aa	1.5±0.5 ABa	2.0±1.2 Ba	2.8±1.3 Aa	1.7±0.6 BCa	4.0±1.6 Aa	2.8±0.7 Ba
**2-way ANOVA** Field: $F_{6,245} = 10.7726$, $P < 0.0001$; Date: $F_{6,245} = 14.9549$, $P < 0.0001$; Field*Date: $F_{36,245} = 2.0869$, $P = 0.0006$							
**Arachnid predators**							
**BS**	0.5±0.2 Aa	0.0±0.0 Ca	0.7±0.2 Aa	0.0±0.0 Aa	0.3±0.2 Aa	0.7±0.3 Aa	0.3±0.2 Aa
**WV**	0.2±0.2 Aab	1.0±0.3 ABa	0.0±0.0 Ab	0.0±0.0 Ab	0.2±0.2 Aab	0.3±0.2 Aab	0.5±0.3 Aab
**SG**	0.0±0.0 Aa	0.3±0.2 ABCa	0.0±0.0 Aa	0.0±0.0 Aa	0.2±0.2 Aa	0.0±0.0 Aa	0.5±0.2 Aa
**OR**	0.3±0.2 Aa	0.3±0.2 ABCa	0.0±0.0 Aa	0.0±0.0 Aa	0.3±0.2 Aa	1.0±0.4 Aa	0.5±0.3 Aa
**SV**	0.7±0.3 Aa	1.2±0.4 Aa	0.2±0.2 Aa	0.2±0.2 Aa	1.3±0.6 Aa	0.3±0.2 Aa	0.7±0.3 Aa
**RO(ORG)**	0.3±0.2 Aab	0.2±0.2 BCb	0.3±0.2 Aab	0.2±0.2 Ab	0.5±0.2 Aab	0.5±0.3 Aab	1.7±0.5 Aa
**RO(IPM)**	0.5±0.3 Aa	0.0±0.0 Ca	0.3±0.2 Aa	0.3±0.2 Aa	0.3±0.2 Aa	0.7±0.4 Aa	1.3±0.6 Aa
**2-way ANOVA** Field: $F_{6,245} = 2.5293$, $P = 0.0215$; Date: $F_{6,245} = 4.661$, $P = 0.0002$; Field*Date: $F_{36,245} = 1.5589$, $P = 0.0278$							
**Insect predators**							
**BS**	0.5±0.2 Aa	0.5±0.3 ABa	1.0±0.4 Aa	0.3±0.3 Aa	0.5±0.3 Aa	0.3±0.2 Ba	0.3±0.3 Aa
**WV**	0.0±0.0 Aa	0.7±0.3 ABa	0.7±0.2 Aa	0.8±0.4 Aa	0.8±0.3 Aa	0.5±0.2 ABa	0.0±0.0 Aa
**SG**	0.3±0.2 Aa	1.8±0.5 ABa	0.8±0.5 Aa	0.8±0.5 Aa	0.7±0.3 Aa	0.5±0.2 ABa	0.7±0.2 Aa
**OR**	0.8±0.5 Aa	0.3±0.2 Ba	0.7±0.3 Aa	1.5±0.8 Aa	0.7±0.5 Aa	1.2±0.3 ABa	0.2±0.2 Aa
**SV**	0.3±0.2 Aa	2.0±0.4 Aa	1.7±1.0 Aa	1.2±0.6 Aa	1.7±0.8 Aa	0.7±0.3 ABa	0.3±0.3 Aa
**RO(ORG)**	0.2±0.2 Ab	0.5±0.2 ABab	1.3±0.5 Aab	1.7±0.3 Aab	1.2±0.3 Aab	2.5±0.7 Aa	1.0±0.6 Aab
**RO(IPM)**	0.2±0.2 Abc	0.8±0.4 ABabc	0.0±0.0 Ac	1.8±0.4 Aa	1.0±0.4 Aabc	1.5±0.3 ABab	1.8±0.8 Aab
**2-way ANOVA** Field: $F_{6,245} = 2.9801$, $P = 0.0079$; Date: $F_{6,245} = 4.0933$, $P = 0.0006$; Field*Date: $F_{36,245} = 1.7041$, $P = 0.0104$							

BS: bare soil; WV: weed vegetation; RO(ORG): rosemary hedgerow (organic orchard); RO(IPM): rosemary hedgerow (IPM orchard); OR: oregano hedgerow; SG: sage hedgerow; SV: savory hedgerow. Capital letters indicate significant differences between orchards while small letters indicate significant differences between assessment dates.
